# Lung Cancer Risk Prediction in Patients with Persistent Pulmonary Nodules Using the Brock Model and Sybil Model

**DOI:** 10.3390/cancers17091499

**Published:** 2025-04-29

**Authors:** Hui Li, Morteza Salehjahromi, Myrna C. B. Godoy, Kang Qin, Courtney M. Plummer, Zheng Zhang, Lingzhi Hong, Simon Heeke, Xiuning Le, Natalie Vokes, Bingnan Zhang, Haniel A. Araujo, Mehmet Altan, Carol C. Wu, Mara B. Antonoff, Edwin J. Ostrin, Don L. Gibbons, John V. Heymach, J. Jack Lee, David E. Gerber, Jia Wu, Jianjun Zhang

**Affiliations:** 1 Department of Thoracic/Head and Neck Medical Oncology, The University of Texas MD Anderson Cancer Center, Houston, TX 77030, USA; hli27@mdanderson.org (H.L.); kqin@mdanderson.org (K.Q.); cmplummer@mdanderson.org (C.M.P.); zzhang11@mdanderson.org (Z.Z.); lhong@mdanderson.org (L.H.); sheeke@mdanderson.org (S.H.); xle1@mdanderson.org (X.L.); nvokes@mdanderson.org (N.V.); bzhang7@mdanderson.org (B.Z.); haaraujo@mdanderson.org (H.A.A.); maltan@mdanderson.org (M.A.); dlgibbon@mdanderson.org (D.L.G.); jheymach@mdanderson.org (J.V.H.); 2 Department of Imaging Physics, The University of Texas MD Anderson Cancer Center, Houston, TX 77030, USA; msalehjahromi@mdanderson.org; 3 Department of Thoracic Imaging, The University of Texas MD Anderson Cancer Center, Houston, TX 77030, USA; mgodoy@mdanderson.org; 4 Department of Genomic Medicine, The University of Texas MD Anderson Cancer Center, Houston, TX 77030, USA; ccwu1@mdanderson.org; 5 Department of Thoracic and Cardiovascular Surgery, The University of Texas MD Anderson Cancer Center, Houston, TX 77030, USA; mbantonoff@mdanderson.org; 6 Department of General Internal Medicine, The University of Texas MD Anderson Cancer Center, Houston, TX 77030, USA; ejostrin@mdanderson.org; 7 Department of Pulmonary Medicine, The University of Texas MD Anderson Cancer Center, Houston, TX 77030, USA; 8 Department of Molecular and Cellular Oncology, The University of Texas MD Anderson Cancer Center, Houston, TX 77030, USA; 9 Department of Biostatistics, The University of Texas MD Anderson Cancer Center, Houston, TX 77030, USA; jjlee@mdanderson.org; 10 Harold C. Simmons Comprehensive Cancer Center, UT Southwestern Medical Center, Dallas, TX 75390, USA; david.gerber@utsouthwestern.edu

**Keywords:** lung cancer risk assessment, persistent pulmonary nodules, brock model, sybil model, precancer interception

## Abstract

Background/Objectives: Persistent pulmonary nodules are at higher risk of developing into lung cancers. Assessing their future cancer risk is essential for successful interception. We evaluated the performance of two risk prediction models for persistent nodules in hospital-based cohorts: the Brock model, based on clinical and radiological characteristics, and the Sybil model, a novel deep learning model for lung cancer risk prediction. Methods: Patients with persistent pulmonary nodules—defined as nodules detected on at least two computed tomography (CT) scans, three months apart, without evidence of shrinkage—were included in the retrospective (*n* = 130) and prospective (*n* = 301) cohorts. We analyzed the correlations between demographic factors, nodule characteristics, and Brock scores and assessed the performance of both models. We also built machine learning models to refine the risk assessment for our cohort. Results: In the retrospective cohort, Brock scores ranged from 0% to 85.82%. In the prospective cohort, 62 of 301 patients were diagnosed with lung cancer, displaying higher median Brock scores than those without lung cancer diagnosis (18.65% vs. 4.95%, *p* < 0.001). Family history, nodule size ≥10 mm, part-solid nodule types, and spiculation were associated with the risks of lung cancer. The Brock model had an AUC of 0.679, and Sybil’s AUC was 0.678. We tested five machine learning models, and the logistic regression model achieved the highest AUC at 0.729. Conclusions: For patients with persistent pulmonary nodules in real-world cancer hospital-based cohorts, both the Brock and Sybil models had values and limitations for lung cancer risk prediction. Optimizing predictive models in this population is crucial for improving early lung cancer detection and interception.

## Simple Summary

The applicability of existing lung cancer risk prediction models, such as the Brock and Sybil frameworks, in hospital-based cohorts with incidentalomas remains underexplored. Persistent pulmonary nodules carry a significant malignancy risk but are often overlooked by current models. In this study, we evaluated the performance of Brock and Sybil models in patients with persistent lung nodules. While both models provided predictive value, they demonstrated limitations in this setting. Our analysis compared multiple machine learning models and found that a logistic regression model outperformed both, offering superior predictive accuracy. These findings suggest that current models for lung cancer risk prediction may require recalibration or the development of new approaches specifically tailored to patients with persistent pulmonary nodules. Enhancing predictive performance in this population is crucial for optimizing clinical decision-making and improving early lung cancer detection and interception.

## 1. Introduction

Lung cancer remains the leading cause of cancer-related mortality [1]. The early detection of lung cancer is crucial for improving patient survival [2]. Persistent or growing pulmonary nodules can be precursors of lung adenocarcinomas (LUADs), including atypical adenomatous hyperplasia (AAH), adenocarcinoma in situ (AIS), and minimally invasive adenocarcinoma (MIA) [3,4,5,6]. Although these precursors can be removed surgically, operative risks and multifocal disease limit this option [7,8]. As an alternative, interception, which uses medications to prevent the progression from precancer to invasive cancer, may be an appealing option [9].

The accurate risk assessment of pulmonary nodules is critical for identifying precancerous lesions and successful precancer interception. The Brock model, also called the Pan-Canadian Early Detection of Lung Cancer risk model, calculates lung cancer likelihood within two to four years using various nodule characteristics and participant variables [10]. It has been recommended by the British Thoracic Society guidelines in clinical practice [11]. Lung cancer interception trials, including Can-Prevent-Lung (NCT04789681) and IMPRINT-Lung (NCT03634241), also incorporated the Brock model in their inclusion criteria. Studies found that the Brock model outperformed or demonstrated similar performance to Lung-RADS in predicting malignant pulmonary nodules [12,13,14,15]. However, the performance of the Brock malignancy risk scoring system has exhibited variability when applied to different populations [16,17,18].

In addition to traditional machine learning models, advancements in artificial intelligence have led to the use of deep learning algorithms for building cancer prediction models. Among these innovative models, the Sybil model stands out as a novel approach designed to predict lung cancer risk directly from a single low-dose computed tomography (LDCT) scan, independent of clinical or demographic data. This model can predict an individual’s future lung cancer risk for up to 6 years, achieving area under the curve (AUC) values ranging as high as 0.96 [19].

Both the Brock model and Sybil model were developed using population-based lung cancer screening data from heavy smokers. However, nearly half of lung cancer cases were diagnosed in never or light smokers who did not meet the lung cancer screening criteria [20]. For these never or light smokers with persistent pulmonary nodules detected on a chest CT for different reasons, predicting lung cancer risk is also important. However, there is limited evidence regarding the performance of Brock or Sybil models in real-world hospital-based cohorts for patients with incidental pulmonary nodules.

With the increasing adaptation of CT-guided lung cancer screening and the advent of chest CT scans for multiple medical reasons, there has been a dramatic increase in the detection of pulmonary nodules. About 50~80% of newly detected nodules resolve during follow-up [21,22], suggesting that a major proportion of these pulmonary nodules are benign processes. On the other hand, persistent or growing lung nodules may carry significant lung cancer risk [23]. Therefore, the risk prediction of persistent pulmonary nodules is more clinically relevant. However, Brock or Sybil models have not been systemically tested in this setting. In this study, we assessed the performance of the Brock model and Sybil models in real-world, cancer hospital-based cohorts of persistent pulmonary nodules. We also attempted to develop prediction models to investigate the potential for improving predictive accuracy in our cohorts.

## 2. Materials and Methods

### 2.1. Study Cohorts

We oversaw the enrollment of a retrospective cohort and a prospective cohort of patients treated at MD Anderson Cancer Center, collecting clinical data such as age, gender, race, ethnicity, smoking history, and family history of lung cancer. The Institutional Review Board (IRB) of MD Anderson Cancer Center approved this study, and informed consent was obtained from all patients.

For the retrospective cohort, we included patients treated between December 2007 and January 2023 who met the following criteria: (1) a pathological diagnosis of lung cancer and (2) available computed tomography (CT) or positron emission tomography/computed tomography (PET/CT) scans taken at least one year before the lung cancer diagnosis. A total of 130 patients met these criteria. All CT or PET/CT scans were examined to determine when lung nodules were first detectable. Follow-up scans taken at least three months later were evaluated to confirm the presence of persistent pulmonary nodules, defined as nodules that showed no shrinkage for at least three months. These nodules were assessed using the Brock model.

For the prospective cohort, we enrolled patients monitored for pulmonary nodules at the MD Anderson Cancer Center between November 2018 and December 2022. Inclusion criteria included persistent pulmonary nodules, defined as nodules showing no shrinkage for at least three months on CT or PET/CT scans. Patients were followed routinely, with intervals determined by their providers. Highly suspicious lung nodules detected during follow-up were biopsied, and the pathological results were reviewed.

### 2.2. Radiologic Assessment

CT or PET/CT scans were reviewed by experienced chest radiologists, who assessed persistent pulmonary nodules. Radiologists measured the maximum diameter and recorded characteristics, including the nodule type (ground-glass opacity, part-solid, or solid types), location, nodule count, emphysema presence, and spiculation, which are all essential for Brock model analysis. In the prospective cohort, exclusion criteria for persistent nodules included (1) metastasis, (2) benign patterns such as inflammation, fibrosis, or calcified granulomas, (3) lesions too small to evaluate, and (4) large or rapidly growing lesions highly suspicious of lung cancer. In cases with multiple nodules, we selected the nodule with the highest Brock score as the primary lesion of interest for follow-up.

### 2.3. Performance of Brock Model and Sybil Model

Four models (1a, 2a, 1b, 2b) were developed using Pan-Canadian Early Detection of Lung Cancer Study (PanCan) data validated with British Columbia Cancer Agency (BCCA) data. Among these, the Brock full model (Model 2b, incorporating spiculation) was used as the primary risk assessment tool for persistent pulmonary nodules [10]. Brock risk scores were recorded at baseline, and the nodules with the highest Brock scores were the primary focus of the analysis. Sybil model risk scores were calculated using the published algorithm [19]. We generated Sybil predictions using the official, publicly released model from MIT/MGH. This package includes five pre-trained Sybil networks, which were used exactly as provided, without any additional training or tuning. No external image preprocessing was necessary, as the Sybil model internally performs Hounsfield unit conversion, intensity clipping, and normalization.

We evaluated the discrimination of the Brock and Sybil models using receiver-operating characteristic (ROC) curves, with the AUC and associated confidence intervals (CIs) representing performance. AUC values ranged from 0 to 1, with higher values indicating better discrimination. Calibration was assessed using calibration plots, comparing predicted probabilities against observed outcomes. Well-calibrated models aligned closely with the diagonal line, reflecting strong agreement between the predictions and actual outcomes.

### 2.4. Development of Machine Learning Models

In our study, we developed five machine learning models, including logistic regression (LR), artificial neural networks (ANNs), eXtreme Gradient Boosting (XGBoost), random forest (RF), and support vector machine (SVM), based on data from our prospective cohort. The features used were consistent with those of the Brock full model. All the models were trained and tested using the dataset split into 70% for training and 30% for testing through random sampling. We employed 5-fold cross-validation during the training process to enhance the robustness of the model and reduce the risk of overfitting. The average performance across these folds was used to evaluate and optimize the model prior to testing on the independent testing set. Subsequently, we evaluated the performance of the models on the testing dataset by calculating the AUC from the ROC analysis. The optimal model was selected based on its performance metrics. The DeLong test was used to compare the performance between the different models [24].

### 2.5. Statistical Analysis

For the retrospective cohort, nodules were categorized based on Brock risk scores (high risk: ≥10% likelihood of lung cancer; low risk: <10%), and comparisons were made between the two risk groups. The 10% cut-off was consistent with established guidelines and other studies [11,18,25]. In the prospective cohort, clinicopathological variables and nodule characteristics were compared between lung cancers and persistent pulmonary nodules, which were not diagnosed as lung cancer. Statistical tests such as Student’s *t*-test, the Mann–Whitney U test, the chi-square test, or Fisher’s exact test were appropriately employed. Multivariate analysis was performed using logistic regression models. The odds ratios (ORs) and 95% confidence intervals (95% CIs) were calculated.

Furthermore, we obtained the AUC from the ROC analysis to assess the performance of the Brock model and Sybil model. AUC values were also utilized to compare different machine learning algorithms. The optimal cut-offs of the models were achieved. We also examined metrics such as sensitivity, specificity, positive predictive value (PPV), and negative predictive value (NPV). All data analyses were performed using R (V 4.2.1, R Foundation for Statistical Computing, Vienna, Austria) and GraphPad Prism 10.0.3 (GraphPad Software, San Diego, CA, USA) software. Statistical significance was defined as a two-sided *p*-value < 0.05.

## 3. Results

### 3.1. Lung Cancer Risk Prediction of Persistent Lung Nodules by Brock Model

To test the performance of the Brock model in predicting the lung cancer risk of persistent lung nodules, we first analyzed a retrospective cohort of 130 patients who presented with persistent pulmonary nodules before lung cancer diagnosis. Among them, 107 (82.3%) were diagnosed with lung adenocarcinoma (LUAD), while 17 (13.1%) had lung squamous cell carcinoma (LUSC), and 6 (4.6%) had other histology types. Using the Brock model, the predicted lung cancer risk scores on the second available CT scan (confirming nodule persistency) ranged from 0% to 85.8%, with a median time of 561.5 days between persistent pulmonary nodule detection and lung cancer diagnosis. Surprisingly, 51 patients (39.2%) had predicted risk scores < 10% (Table 1).

**Figure 1:**
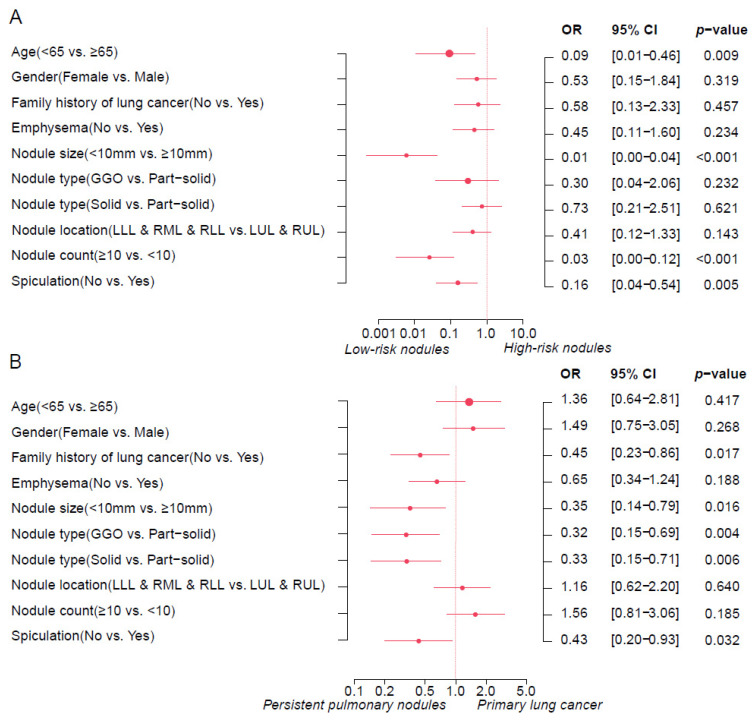
Multivariate logistic regression analysis. (**A**) This forest plot identifies variables associated with high and low Brock risk scores in the retrospective cohort. (**B**) The forest plot displays variables associated with primary lung cancer and persistent pulmonary nodules in the prospective cohort. The effect estimates were the odds ratios (ORs). Error bars represent 95% CIs, indicating the precision of the OR estimates. *p*-values were calculated to determine the statistical significance of each predictor’s association with cancer risk scores or primary lung cancer. The size of the dots indicates the study effect size. GGO: ground-glass opacity; LUL: left upper lobe; RUL: right upper lobe; LLL: left lower lobe; RML: right middle lobe; RLL: right lower lobe.

In univariate analysis, low risk was associated with a nodule size < 10 mm (51% vs. 3.8%, *p* < 0.001), ground-glass opacity (GGO) (25.5% vs. 8.9%, *p* < 0.001), nodule count ≥ 10 (62.7% vs. 30.4%, *p* < 0.001), and a lack of spiculation (72.5% vs. 24.1%, *p* < 0.001). Interestingly, patients with adenocarcinoma histology were more likely to exhibit high-risk scores (70.6% vs. 89.9%, *p* = 0.008). However, age, gender, smoking history, a family history of lung cancer, emphysema, and nodule location were not significantly associated with lung cancer risk. Multivariate logistic regression analysis further revealed that patients younger than 65 years (OR: 0.09, 95% CI: 0.01–0.46, *p* = 0.009) with a nodule size < 10 mm (OR: 0.01, 95% CI: 0.00–0.04, *p* < 0.001), nodule count ≥ 10 (OR: 0.03, 95%CI: [0.00–0.12], *p* < 0.001), and a lack of spiculation (OR: 0.16, 95% CI: 0.04–0.54, *p* = 0.005) were more likely to have low-risk nodules (Figure 1A). These observations suggest that the Brock model may underestimate the lung cancer risk of persistent nodules.

Next, we applied the Brock model to a prospective cohort of 301 patients with persistent pulmonary nodules (Table 2). The median follow-up time was 584 days from the detection of persistent pulmonary nodules to the time of data lock. In this cohort, 62 of 301 patients (20.6%) were diagnosed with lung cancer, and the median time between the persistent pulmonary nodule detection and lung cancer diagnosis was 489 days. For the remaining 239 patients who were not diagnosed with lung cancer, the median follow-up time was 588 days.

In the univariate analysis, a family history of lung cancer (45.2% vs. 27.6%, *p* = 0.014), emphysema (54.8% vs. 34.7%, *p* = 0.006), nodule size ≥ 10 mm(87.1% vs. 61.5%, *p* < 0.001), part-solid nodules (compared to solid and GGO types, 51.6% vs. 22.2% vs. 25.8, *p* < 0.001), and spiculation (33.9% vs. 15.5%, *p* = 0.002) were associated with primary lung cancer. However, no significant correlations were observed between primary lung cancer and other persistent pulmonary nodules in terms of age, gender, smoking history, or nodule location (Table 2). In the multivariate analysis, independent risk factors associated with primary lung cancer included family history of lung cancer (*p* = 0.017), nodule size ≥ 10 mm (*p* = 0.016), part-solid nodule types (*p* = 0.004 vs. GGO, *p* = 0.006 vs. solid nodules), and spiculation (*p* = 0.032) (Figure 1B).

**Figure 2:**
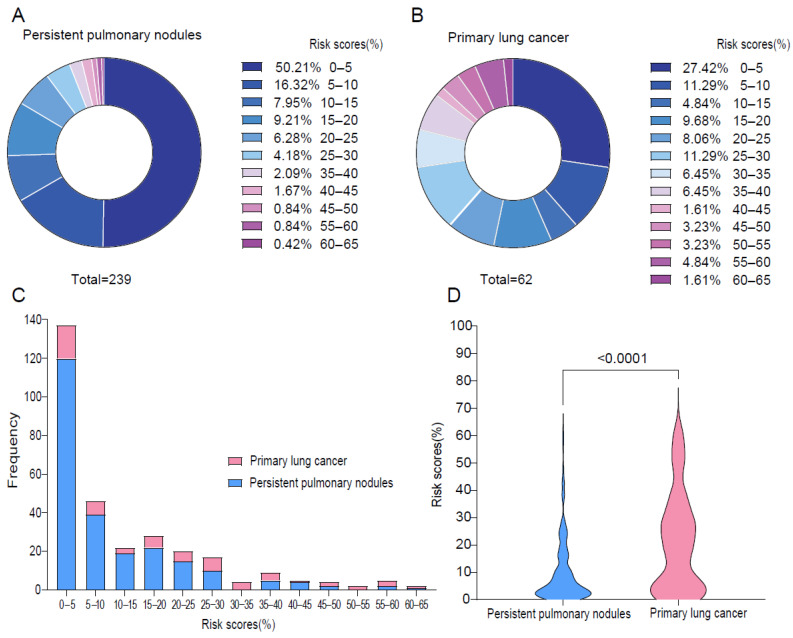
The distribution of Brock risk scores in patients with persistent pulmonary nodules and primary lung cancer in the prospective cohort. (**A**,**B**): The donut charts depict the proportion of different risk scores among patients with persistent pulmonary nodules and primary lung cancer, respectively. (**C**): The bar plot showed the frequency of patients with persistent pulmonary nodules and primary lung cancer, categorized by different risk scores. (**D**): The violin plot highlights the differences in risk score distributions between the lung nodules and primary lung cancer, with a noted significance level (*p* < 0.001).

**Figure 3:**
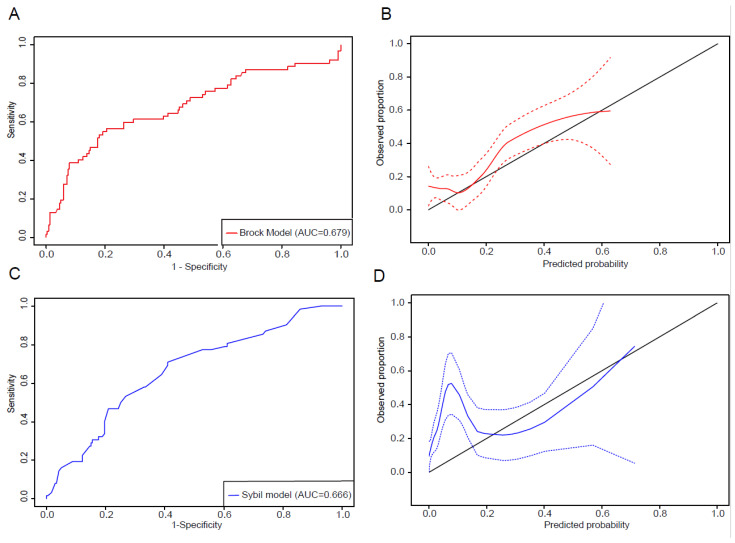
The assessment of the Brock model and Sybil model in the prospective cohort. (**A**) Evaluation of the Brock model’s discrimination ability using the ROC curve. (**B**) Calibration assessment of the Brock model using a calibration plot. The calibration curve (red solid line) represents the relationship between predicted and observed probabilities, with 95% confidence intervals shown as red dashed lines. The black diagonal line represents the ideal calibration line, where predicted probabilities perfectly match observed outcomes. (**C**) Evaluation of the Sybil model’s discrimination ability using ROC curves for 1-year risk scores. (**D**) Calibration assessment of the Sybil model based on 1-year risk scores using a calibration plot. The calibration curve (blue solid line) represents the relationship between predicted and observed probabilities, with 95% confidence intervals shown as blue dashed lines. The black diagonal line represents the ideal calibration line, where predicted probabilities perfectly match observed outcomes.

Notably, among patients without a cancer diagnosis, 33.47% had Brock risk scores ≥ 10%, and among lung nodules with a lung cancer diagnosis, 38.71% had risk scores < 10% (Figure 2A,B). Furthermore, among the 183 patients with risk scores < 10%, 24 (13.11%) were diagnosed with lung cancer (Figure 2C). The median risk score was 18.65% (Q1–Q3: 4.42–31.69%) for lung nodules with primary lung cancer diagnosis compared to 4.95% (Q1–Q3: 2.06–15.28%) for the persistent pulmonary nodules without lung cancer diagnosis during the follow-up period (*p* < 0.001, Figure 2D). Using the ROC analysis, the Brock model yielded an AUC of 0.679 (95% CI: 0.595–0.763, Figure 3A). The optimal cut-off value on the ROC curve was 0.169, and the sensitivity, specificity, positive predictive value, and negative predictive value were 0.565, 0.795, 0.417, and 0.874, respectively. These observations indicate that the Brock model, which was trained on low-dose CT scans in heavy smokers, can predict cancer risk but has important limitations for the lung cancer risk prediction of persistent lung nodules in the hospital setting. The calibration plot shows that the most observed proportions were above the diagonal line, which suggests the underestimation of lung cancer (Figure 3B).

### 3.2. Assessment of Sybil Model Performance

Next, we applied the Sybil model to the prospective cohort to assess the risk prediction performance of the Sybil model for persistent lung nodules in this hospital-based cohort. The median 1-year Sybil risk score was 0.031 for patients who were diagnosed with lung cancer and 0.011 for those without lung cancer diagnosis (*p* < 0.001). Predicted cancer risks for years 2–6 were also significantly higher in patients with a lung cancer diagnosis (*p* < 0.001, Table 3) than those without. The ROC analysis showed an AUC of 0.666 for 1-year lung cancer risk (95% CI: 0.597–0.740, Figure 3C). The calibration plot revealed an underestimation of cancer risk for low-risk nodules (above the diagonal line) and an overestimation for high-risk nodules (below the diagonal line, Figure 3D). The C-index for 6-year cancer risk was 0.641 (95% CI: 0.565–0.718).

**Figure 4:**
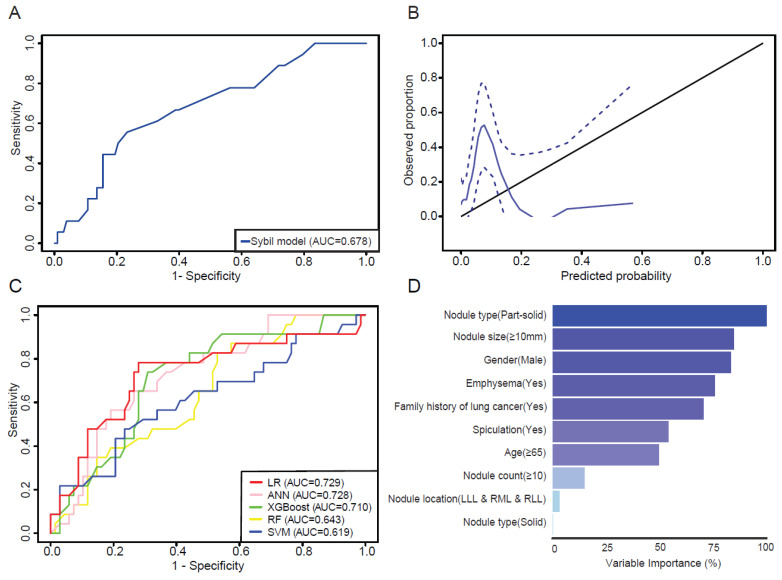
The assessment of the Sybil model in a sub-cohort of patients (N = 121) undergoing non-contrast-enhanced CT scans and comparison of different machine learning models in the prospective cohort. (**A**) An evaluation of the Sybil model’s discrimination ability using ROC curves for the 1-year risk score. (**B**) Calibration assessment of the Sybil model based on 1-year risk scores using a calibration plot. The calibration curve (blue solid line) represents the relationship between predicted and observed probabilities, with 95% confidence intervals shown as blue dashed lines. The black diagonal line represents the ideal calibration line, where predicted probabilities perfectly match observed outcomes. (**C**) Comparison of the performance of five different machine learning models in the testing cohort, depicted by ROC curves. (**D**) The feature importance plot for the logistic regression (LR) model in the training cohort. The variable importance values range from 0% to 100%. The most important variable has a relative importance of 100%. LR: logistic regression; ANN: artificial neural network; XGBoost: eXtreme Gradient Boosting; RF: random forest; SVM: support vector machine; LLL: left lower lobe; RML: right middle lobe; RLL: right lower lobe.

The original Sybil model was built using non-contrast-enhanced LDCT; however, our cohort included 167 patients with contrast-enhanced CT scans and 14 with PET/CT scans, which may have contributed to the observed suboptimal performance. To address this, a subgroup analysis of patients (N = 121) with only non-contrast-enhanced CT scans was conducted (Table 4), revealing an AUC of 0.678 (95% CI: 0.591–0.740) for 1-year lung cancer risk, indicating only a modest improvement in performance (Figure 4A). The calibration plot for this subgroup showed a similar pattern (Figure B). To further investigate whether contrast-enhanced CT or PET/CT imaging affects the performance of the Sybil model, we conducted a subgroup analysis. Violin plots showed no significant differences in 1-year risk score distributions between the CT and PET/CT subgroups, nor between contrast-enhanced and non-contrast CT subgroups. ROC curve analysis further evaluated the model’s discrimination ability, showing that CT (AUC 0.663) and PET/CT (AUC 0.656) demonstrated comparable performance. Non-contrast CT (AUC 0.688) had a slightly higher AUC than contrast-enhanced CT (AUC 0.651), suggesting that contrast administration had a minimal impact on model performance. These results are presented in Supplementary Materials, Figure S1. Taken together, these results suggest that Sybil models can predict cancer risk in this hospital-based cohort but with limitations.

### 3.3. Evaluation of Machine Learning Models

Given the limited performance of Brock and Sybil models in the hospital-based cohort, we attempted to construct machine learning models utilizing the same variables as those in the Brock model, which led to the development of five machine learning models (Figure 4C). The AUCs of the LR, ANN, XGBoost, RF, and SVM models were 0.729 (95% CI: 0.597–0.861), 0.728 (95% CI: 0.615–0.841), 0.710 (95% CI: 0.593–0.828), 0.643 (95% CI: 0.520–0.765), and 0.619 (95% CI: 0.478–0.760), respectively. Among these models, the logistic regression model had the best performance, achieving an AUC of 0.729. The optimal cut-off value for the LR model was 0.177. At this threshold, the LR model showed a sensitivity of 0.782, specificity of 0.721, positive predictive value of 0.487, and negative predictive value of 0.907. Additionally, we identified key features that significantly influenced the predictive capacity of the LR model. These included part-solid nodule types (100.00%), nodule size ≥ 10 mm (84.71%), male gender (83.26%), the presence of emphysema (75.89%), a family history of lung cancer (70.69%) and others. The nodule count and nodule location play a lesser role in the construction of the LR model (Figure 4D).

To further evaluate model performance, we compared clinically relevant metrics (sensitivity, specificity, PPV, and NPV) across the logistic regression (LR) model, the Brock model, and the Sybil model at various decision thresholds, as presented in Supplementary Table S1. The Brock model demonstrated very high sensitivity but extremely low specificity, limiting its utility in reducing false positives. The Sybil model showed strong specificity, indicating its potential value in accurately identifying non-cancer cases and minimizing unnecessary follow-up. However, its sensitivity declined substantially at higher thresholds, raising concerns about the potential under-detection of lung cancer cases. In comparison, the LR model provided the most balanced performance across thresholds, achieving higher PPV and consistently strong NPV, suggesting improved overall discrimination for persistent pulmonary nodules.

## 4. Discussion

High-risk pulmonary nodules, which may represent lung cancer precursors, necessitate vigilant monitoring and timely intervention to prevent malignant progression [26]. Assessing future cancer risk is a critical strategy for identifying precancerous lesions at risk of malignant progression. Among the various models used for cancer risk prediction, the Brock model and Sybil model have shown strong performance in lung cancer screening cohorts [10,19]. As both models were developed based on screening cohorts (heavy smokers and low-dose CT images), whether these models are applicable to patients with nodules identified in non-screening settings is unclear. In addition, many pulmonary nodules fluctuate or disappear to follow-up. Studies found that 69.8% of the part-solid nodules at the screening CT were transient [22], while 75% of the persistent GGO nodules were attributed to early lung cancer [23]. Thus, we focused on persistent lung nodules in the current study. To the best of our knowledge, this study is the first to evaluate the performance of the Brock and Sybil models for persistent pulmonary nodules. In these hospital-based non-screening cohorts of persistent pulmonary nodules, both Brock and Sybil models demonstrated value in predicting future lung cancer risks but with obvious limitations.

For the Brock model, ROC curve analysis in our prospective cohort yielded an AUC of 0.689. While this performance is lower compared to the original study [10] and subsequent validation studies [16,18,27], it remains reasonable given the small sample size, the selection of persistent nodules, and the non-screening clinical setting in the current study. However, in both retrospective and prospective cohorts with persistent lung nodules, nearly 40% of patients with confirmed lung cancer had a Brock risk score of <10%, indicating an underestimation of future lung cancer risks in these hospital-based cohorts. Conversely, 33.47% of patients without a lung cancer diagnosis (noting the limitation of a relatively short follow-up period) had a Brock risk score > 10%, suggesting the potential overestimation of short-term lung cancer risk in this context. Importantly, factors such as age, nodule size, nodule type (GGO versus partial solid), and the presence of spiculation were found to be associated with lung cancer risk, which is consistent with the Brock model. This supports the notion that these clinical and radiologic features hold significant value for lung cancer risk prediction, even in hospital-based cohorts, and highlights their relevance for future risk model development in this clinical setting.

With advancements in artificial intelligence (AI), deep learning algorithms have been used to develop models for predicting lung cancer risk, primarily in screening settings, including the Sybil model. Many of these models have demonstrated better performance than the Brock model. For example, the LCP-CNN achieved an AUC of 0.936 compared to the Brock model’s AUC of 0.873 [28]. Another deep learning model outperformed the full Brock model in both the DLCST cohort (AUC 0.97 vs. 0.94) and the Multicentric Italian Lung Detection Trial (MILD) cohort (AUC 0.99 vs. 0.96) [29]. The Sybil model is a novel deep learning approach that relies solely on CT scans without requiring clinical variables, making it easier to implement in clinical practice. In the current study on persistent lung nodules in a non-screening setting, the Sybil model achieved a clear distinction between nodules that were confirmed to be lung cancer and those that were not. The Sybil model achieved an AUC of 0.666 for 1-year lung cancer risk and a C-index of 0.641 for over 6 years. However, the calibration plot revealed both the underestimation and overestimation of lung cancer risk. Focusing on non-contrast CT scans in our cohort, the AUC slightly improved to 0.678, suggesting that different image modalities only have some impact on the predictive performance of Sybil and AI models developed in screening settings and may not be suitable for hospital-based cohorts.

Our findings demonstrate both the value and the limitations of the Brock and Sybil models for persistent pulmonary nodules detected in hospital-based cohorts. Therefore, novel models are needed for the identification of persistent pulmonary nodules in a non-screening setting for lung cancer risk prediction. Several studies have developed models to improve lung cancer risk prediction in hospital-based cohorts. For instance, the Thoracic Research Evaluation and Treatment (TREAT) model, using logistic regression with variables like body mass index (BMI), nodule growth, prior cancer history, and positive fluorodeoxyglucose-PET (FDG-PET) scan findings, achieved an AUC of 0.85 compared to Brock’s AUC of 0.68 [30]. A random forest model integrating 19 variables, including blood tests and lifestyle factors, reached an AUC of 0.851 versus Brock’s AUC of 0.575 [31]. The PKU-M model, leveraging XGBoost, achieved an AUC of 0.909 versus Brock’s AUC of 0.806 [32]. Deep learning models and radiomics-based approaches have also been explored in clinical settings, demonstrating superior performance [33,34]. With early promise from these models, further validation is required to ensure their effectiveness and applicability across diverse cohorts. So far, none of these models have been applied to predict the lung cancer risks of persistent pulmonary nodules. In this study, we also explored five different machine learning algorithms for the lung cancer risk prediction of persistent lung nodules in the clinical-based setting and demonstrated an improvement in risk prediction.

This study has several limitations that should be acknowledged. First, the cohorts included a relatively small number of patients, which may limit the statistical power and generalizability of the findings. Second, the median follow-up duration of the prospective cohort may be insufficient to capture all incident lung cancers, particularly indolent cases that manifest over longer timeframes. Third, this study was conducted at a single institution, and external validation in independent, multi-institutional cohorts is needed to confirm the model’s robustness and generalizability across diverse clinical settings. Future work will focus on refining the model and validating its clinical utility in broader populations.

Our study has several notable strengths. First, we conducted a comprehensive evaluation of the performance of both the Brock model, a traditional machine learning approach, and the Sybil model, a novel AI-based model, using two distinct cohorts. This design enabled us to investigate whether models developed in screening settings could be effectively applied to clinical cohorts while also identifying factors influencing their performance. Second, we explored five machine learning methods and identified the LR model as the most effective one with its key features, providing key insights for refining the Brock model and developing future models. Additionally, our study is the first to highlight the importance of persistent pulmonary nodules in improving the detection of early-stage lung cancer. By addressing this research gap, our findings contribute valuable insights into the field of lung cancer early detection and interception, particularly in hospital-based cohorts with incidentally detected pulmonary nodules.

## 5. Conclusions

Although numerous predictive models for lung cancer risk have been developed, their integration into clinical practice remains limited due to challenges such as inadequate external validation, poor integration into clinical workflows, and limited generalizability across diverse patient populations. Our findings revealed variability in the predictions of existing models, emphasizing their limitations in real-world clinical settings. This underscores the need to adapt models like Brock and Sybil for use in clinical cohorts, particularly for nodules detected in non-screening settings. This study also highlights the importance of persistent pulmonary nodules in cancer risk assessment, providing a foundation for future research aimed at developing personalized prevention strategies. To enhance early lung cancer detection, future efforts should focus on creating predictive models that incorporate a broader range of clinical and radiological variables, advanced algorithms, and dynamic changes in persistent nodules. Such approaches could support more effective interception strategies for precancerous lesions.

## Figures and Tables

**Table 1:** Clinicopathological variables and nodule characteristics of the retrospective cohort.

	Overall (N = 130)	Low-Risk Nodules (N = 51)	High-Risk Nodules (N = 79)	*p*-Value
Age				
<65	32 (24.6%)	16 (31.4%)	16 (20.3%)	0.219
≥65	98 (75.4%)	35 (68.6%)	63 (79.7%)	
Gender				
Male	45 (34.6%)	17 (33.3%)	28 (35.4%)	0.954
Female	85 (65.4%)	34 (66.7%)	51 (64.6%)	
Race				
White	108 (83.1%)	41 (80.4%)	67 (84.8%)	0.804 *
Asian	9 (6.9%)	5 (9.8%)	4 (5.1%)	
Black	10 (7.7%)	4 (7.8%)	6 (7.6%)	
Others	3 (2.3%)	1 (2.0%)	2 (2.5%)	
Ethnicity				
Non-Hispanic or Latino	117 (90.0%)	45 (88.2%)	72 (91.1%)	0.818 *
Hispanic or Latino	6 (4.6%)	3 (5.9%)	3 (3.8%)	
Unknown	7 (5.4%)	3 (5.9%)	4 (5.1%)	
Smoking history				
Current	5 (3.8%)	1 (2.0%)	4 (5.1%)	0.754 *
Former	88 (67.7%)	36 (70.6%)	52 (65.8%)	
Never	37 (28.5%)	14 (27.5%)	23 (29.1%)	
Histology				
LUAD	107 (82.3%)	36 (70.6%)	71 (89.9%)	0.008 *
LUSC	17 (13.1%)	10 (19.6%)	7 (8.9%)	
Others	6 (4.6%)	5 (9.8%)	1 (1.3%)	
Family history of lung cancer				
No	94 (72.3%)	34 (66.7%)	60 (75.9%)	0.340
Yes	36 (27.7%)	17 (33.3%)	19 (24.1%)	
Emphysema				
No	88 (67.7%)	35 (68.6%)	53 (67.1%)	1.000
Yes	42 (32.3%)	16 (31.4%)	26 (32.9%)	
Nodule size(mm)				
<10	29 (22.3%)	26 (51.0%)	3 (3.8%)	<0.001 *
≥10	101 (77.7%)	25 (49.0%)	76 (96.2%)	
Nodule type				
GGO	20 (15.4%)	13 (25.5%)	7 (8.9%)	0.032
Part-solid	56 (43.1%)	18 (35.3%)	38 (48.1%)	
Solid	54 (41.5%)	20 (39.2%)	34 (43.0%)	
Nodule location				
LUL & RUL	73 (56.2%)	24 (47.1%)	49 (62.0%)	0.134
LLL & RML & RLL	57 (43.8%)	27 (52.9%)	30 (38.0%)	
Nodule count				
<10	74 (56.9%)	19 (37.3%)	55 (69.6%)	<0.001
≥10	56 (43.1%)	32 (62.7%)	24 (30.4%)	
Nodule spiculation				
No	56 (43.1%)	37 (72.5%)	19 (24.1%)	<0.001
Yes	74 (56.9%)	14 (27.5%)	60 (75.9%)	

LUAD: lung adenocarcinoma; LUSC: lung squamous cell carcinoma; GGO: ground-glass opacity; LUL: left upper lobe; RUL: right upper lobe; LLL: left lower lobe; RML: right middle lobe; RLL: right lower lobe. * *p* values were calculated using Fisher’s exact test.

**Table 2:** Clinicopathological variables and nodule characteristics of the prospective cohort.

	Overall (N = 301)	Persistent Pulmonary Nodules (N = 239)	Primary Lung Cancer (N = 62)	*p*-Value
Age				
<65	76 (25.2%)	61 (25.5%)	15 (24.2%)	0.960
≥65	225 (74.8%)	178 (74.5%)	47 (75.8%)	
Gender				
Male	102 (33.9%)	85 (35.6%)	17 (27.4%)	0.291
Female	199 (66.1%)	154 (64.4%)	45 (72.6%)	
Race				
White	246 (81.7%)	197 (82.4%)	49 (79.0%)	0.338 *
Asian	24 (8.0%)	18 (7.5%)	6 (9.7%)	
Black	18 (6.0%)	12 (5.0%)	6 (9.7%)	
Others	13 (4.3%)	12 (5.0%)	1 (1.6%)	
Ethnicity				
Non-Hispanic or Latino	278 (92.4%)	218 (91.2%)	60 (96.8%)	0.295 *
Hispanic or Latino	17 (5.6%)	16 (6.7%)	1 (1.6%)	
Unknown	6 (2.0%)	5 (2.1%)	1 (1.6%)	
Smoking history				
Current	18 (6.0%)	14 (5.9%)	4 (6.5%)	0.170 *
Former	201 (66.8%)	154 (64.4%)	47 (75.8%)	
Never	82 (27.2%)	71 (29.7%)	11 (17.7%)	
Family history of lung cancer				
No	205 (68.1%)	171 (71.5%)	34 (54.8%)	0.014
Yes	94 (31.2%)	66 (27.6%)	28 (45.2%)	
Missing	2 (0.7%)	2 (0.8%)	0 (0%)	
Emphysema				
No	184 (61.1%)	156 (65.3%)	28 (45.2%)	0.006
Yes	117 (38.9%)	83 (34.7%)	34 (54.8%)	
Nodule size (mm)				
<10	100 (33.2%)	92 (38.5%)	8 (12.9%)	<0.001
≥10	201 (66.8%)	147 (61.5%)	54 (87.1%)	
Nodule type				
GGO	121 (40.2%)	105 (43.9%)	16 (25.8%)	<0.001
Part-solid	85 (28.2%)	53 (22.2%)	32 (51.6%)	
Solid	95 (31.6%)	81 (33.9%)	14 (22.6%)	
Nodule location				
LUL & RUL	164 (54.5%)	131 (54.8%)	33 (53.2%)	0.936
LLL & RML & RLL	137 (45.5%)	108 (45.2%)	29 (46.8%)	
Nodule count				
<10	141 (46.8%)	119 (49.8%)	22 (35.5%)	0.061
≥10	160 (53.2%)	120 (50.2%)	40 (64.5%)	
Nodule spiculation				
No	243 (80.7%)	202 (84.5%)	41 (66.1%)	0.002
Yes	58 (19.3%)	37 (15.5%)	21 (33.9%)	

GGO: ground-glass opacity; LUL: left upper lobe; RUL: right upper lobe; LLL: left lower lobe; RML: right middle lobe; RLL: right lower lobe. * *p* values were calculated using Fisher’s exact test.

**Table 3:** Nodule risk assessment using the Sybil model in the prospective cohort.

	Persistent Pulmonary Nodules (N = 239)	Primary Lung Cancer (N = 62)	*p*-Value
1-year risk			
Mean (SD)	0.0501 (0.0935)	0.0991 (0.138)	<0.001
Median [Min, Max]	0.0109 [0, 0.569]	0.0310 [0.00117, 0.714]	
2-year risk			
Mean (SD)	0.0761 (0.125)	0.144 (0.173)	<0.001
Median [Min, Max]	0.0238 [0, 0.714]	0.0598 [0.00255, 0.824]	
3-year risk			
Mean (SD)	0.0922 (0.131)	0.167 (0.179)	<0.001
Median [Min, Max]	0.0382 [0, 0.744]	0.0852 [0.00783, 0.828]	
4-year risk			
Mean (SD)	0.104 (0.136)	0.181 (0.181)	<0.001
Median [Min, Max]	0.0561 [0, 0.763]	0.0981 [0.0110, 0.851]	
5-year risk			
Mean (SD)	0.116 (0.142)	0.197 (0.189)	<0.001
Median [Min, Max]	0.0683 [0, 0.800]	0.109 [0.0184, 0.869]	
6-year risk			
Mean (SD)	0.151 (0.159)	0.246 (0.206)	<0.001
Median [Min, Max]	0.0971 [0, 0.836]	0.154 [0.0309, 0.882]	
**CT types**			
With contrast	127 (53.1%)	40 (64.5%)	0.143
Without contrast	112 (46.9%)	22 (35.5%)	
**CT or PET/CT**			
CT	230 (96.2%)	57 (91.9%)	0.274
PET/CT	9 (3.8%)	5 (8.1%)	

CT: computed tomography; PET/CT: positron emission tomography/computed tomography.

**Table 4:** **The** Sybil model risk assessment of a sub-cohort of patients undergoing non-contrast-enhanced CT scans.

	Persistent Pulmonary Nodules (N = 103)	Primary Lung Cancer (N = 18)	*p*-Value
1-year risk			
Mean (SD)	0.0382 (0.0789)	0.0731 (0.0957)	0.016
Median [Min, Max]	0.0109 [0, 0.569]	0.0310 [0.00178, 0.352]	
2-year risk			
Mean (SD)	0.0601 (0.105)	0.112 (0.126)	0.025
Median [Min, Max]	0.0238 [0.00157, 0.714]	0.0598 [0.00528, 0.463]	
3-year risk			
Mean (SD)	0.0753 (0.108)	0.134 (0.131)	0.019
Median [Min, Max]	0.0382 [0.00295, 0.744]	0.0852 [0.00904, 0.508]	
4-year risk			
Mean (SD)	0.0873 (0.112)	0.151 (0.137)	0.019
Median [Min, Max]	0.0561 [0.00490, 0.763]	0.0981 [0.0132, 0.530]	
5-year risk			
Mean (SD)	0.0989 (0.118)	0.165 (0.145)	0.019
Median [Min, Max]	0.0683 [0.00836, 0.800]	0.109 [0.0195, 0.573]	
6-year risk			
Mean (SD)	0.132 (0.133)	0.210 (0.161)	0.019
Median [Min, Max]	0.104 [0.0144, 0.836]	0.154 [0.0330, 0.653]	

## Data Availability

The data may be available from the corresponding author based on reasonable request.

## Supplementary Materials

The following supporting information can be downloaded at https://www.mdpi.com/article/10.3390/cancers17091499/s1. Figure S1: Comparison of the Sybil model’s performance across imaging subgroups. (A) Violin plot comparing the distribution of 1-year risk scores between patients who underwent CT versus PET/CT. (B) ROC curves evaluating the Sybil model’s discrimination ability for 1-year risk scores in patients imaged with CT versus PET/CT. (C) Violin plot comparing the distribution of 1-year risk scores between patients who underwent contrast-enhanced CT and those who underwent non-contrast-enhanced CT. (D) ROC curves evaluating the Sybil model’s discrimination ability for 1-year risk scores in patients who received contrast-enhanced CT versus non-contrast-enhanced CT; Table S1: Comparison of sensitivity, specificity, positive predictive value (PPV), and negative predictive value (NPV) across the Brock, Sybil, and logistic regression (LR) models.

## References

[1] Siegel R.L., Giaquinto A.N., Jemal A. (2024). Cancer statistics, 2024. CA A Cancer J. Clin..

[2] Goldstraw P., Chansky K., Crowley J., Rami-Porta R., Asamura H., Eberhardt W.E., Nicholson A.G., Groome P., Mitchell A., Bolejack V. (2016). The IASLC Lung Cancer Staging Project: Proposals for Revision of the TNM Stage Groupings in the Forthcoming (Eighth) Edition of the TNM Classification for Lung Cancer. J. Thorac. Oncol..

[3] Detterbeck F.C., Homer R.J. (2011). Approach to the ground-glass nodule. Clin. Chest Med..

[4] Kodama K., Higashiyama M., Takami K., Oda K., Okami J., Maeda J., Koyama M., Nakayama T. (2008). Treatment strategy for patients with small peripheral lung lesion(s): Intermediate-term results of prospective study. Eur. J. Cardiothorac. Surg..

[5] Mun M., Kohno T. (2007). Efficacy of thoracoscopic resection for multifocal bronchioloalveolar carcinoma showing pure ground-glass opacities of 20 mm or less in diameter. J. Thorac. Cardiovasc. Surg..

[6] Ohtsuka T., Watanabe K., Kaji M., Naruke T., Suemasu K. (2006). A clinicopathological study of resected pulmonary nodules with focal pure ground-glass opacity. Eur. J. Cardiothorac. Surg..

[7] Tomonaga N., Nakamura Y., Yamaguchi H., Ikeda T., Mizoguchi K., Motoshima K., Doi S., Nakatomi K., Iida T., Hayashi T. (2013). Analysis of Intratumor Heterogeneity of EGFR Mutations in Mixed Type Lung Adenocarcinoma. Clin. Lung Cancer.

[8] Nambu A., Araki T., Taguchi Y., Ozawa K., Miyata K., Miyazawa M., Hiejima Y., Saito A. (2005). Focal area of ground-glass opacity and ground-glass opacity predominance on thin-section CT: Discrimination between neoplastic and non-neoplastic lesions. Clin. Radiol..

[9] Blackburn E.H. (2011). Cancer interception. Cancer Prev. Res..

[10] McWilliams A., Tammemagi M.C., Mayo J.R., Roberts H., Liu G., Soghrati K., Yasufuku K., Martel S., Laberge F., Gingras M. (2013). Probability of cancer in pulmonary nodules detected on first screening CT. N. Engl. J. Med..

[11] Callister M.E., Baldwin D.R., Akram A.R., Barnard S., Cane P., Draffan J., Franks K., Gleeson F., Graham R., Malhotra P. (2015). British Thoracic Society guidelines for the investigation and management of pulmonary nodules. Thorax.

[12] van Riel S.J., Ciompi F., Jacobs C., Winkler Wille M.M., Scholten E.T., Naqibullah M., Lam S., Prokop M., Schaefer-Prokop C., van Ginneken B. (2017). Malignancy risk estimation of screen-detected nodules at baseline CT: Comparison of the PanCan model, Lung-RADS and NCCN guidelines. Eur. Radiol..

[13] Kim H., Kim H.Y., Goo J.M., Kim Y. (2021). External validation and comparison of the Brock model and Lung-RADS for the baseline lung cancer CT screening using data from the Korean Lung Cancer Screening Project. Eur. Radiol..

[14] Sundaram V., Gould M.K., Nair V.S. (2021). A Comparison of the PanCan Model and Lung-RADS to Assess Cancer Probability Among People With Screening-Detected, Solid Lung Nodules. Chest.

[15] Hammer M.M., Palazzo L.L., Kong C.Y., Hunsaker A.R. (2019). Cancer Risk in Subsolid Nodules in the National Lung Screening Trial. Radiology.

[16] Winter A., Aberle D.R., Hsu W. (2019). External validation and recalibration of the Brock model to predict probability of cancer in pulmonary nodules using NLST data. Thorax.

[17] Winkler Wille M.M., van Riel S.J., Saghir Z., Dirksen A., Pedersen J.H., Jacobs C., Thomsen L.H., Scholten E.T., Skovgaard L.T., van Ginneken B. (2015). Predictive Accuracy of the PanCan Lung Cancer Risk Prediction Model -External Validation based on CT from the Danish Lung Cancer Screening Trial. Eur. Radiol..

[18] White C.S., Dharaiya E., Campbell E., Boroczky L. (2017). The Vancouver Lung Cancer Risk Prediction Model: Assessment by Using a Subset of the National Lung Screening Trial Cohort. Radiology.

[19] Mikhael P.G., Wohlwend J., Yala A., Karstens L., Xiang J., Takigami A.K., Bourgouin P.P., Chan P., Mrah S., Amayri W. (2023). Sybil: A Validated Deep Learning Model to Predict Future Lung Cancer Risk from a Single Low-Dose Chest Computed Tomography. J. Clin. Oncol..

[20] Taiwo E.O., Yorio J.T., Yan J., Gerber D.E. (2012). How have we diagnosed early-stage lung cancer without radiographic screening? A contemporary single-center experience. PLoS ONE.

[21] Lee C.T. (2015). What do we know about ground-glass opacity nodules in the lung?. Transl. Lung Cancer Res..

[22] Lee S.M., Park C.M., Goo J.M., Lee C.H., Lee H.J., Kim K.G., Kang M.J., Lee I.S. (2010). Transient part-solid nodules detected at screening thin-section CT for lung cancer: Comparison with persistent part-solid nodules. Radiology.

[23] Kim H.Y., Shim Y.M., Lee K.S., Han J., Yi C.A., Kim Y.K. (2007). Persistent pulmonary nodular ground-glass opacity at thin-section CT: Histopathologic comparisons. Radiology.

[24] DeLong E.R., DeLong D.M., Clarke-Pearson D.L. (1988). Comparing the areas under two or more correlated receiver operating characteristic curves: A nonparametric approach. Biometrics.

[25] Chung K., Mets O.M., Gerke P.K., Jacobs C., den Harder A.M., Scholten E.T., Prokop M., de Jong P.A., van Ginneken B., Schaefer-Prokop C.M. (2018). Brock malignancy risk calculator for pulmonary nodules: Validation outside a lung cancer screening population. Thorax.

[26] MacMahon H., Naidich D.P., Goo J.M., Lee K.S., Leung A.N.C., Mayo J.R., Mehta A.C., Ohno Y., Powell C.A., Prokop M. (2017). Guidelines for Management of Incidental Pulmonary Nodules Detected on CT Images: From the Fleischner Society 2017. Radiology.

[27] Nair V.S., Sundaram V., Desai M., Gould M.K. (2018). Accuracy of Models to Identify Lung Nodule Cancer Risk in the National Lung Screening Trial. Am. J. Respir. Crit. Care Med..

[28] Chetan M.R., Dowson N., Price N.W., Ather S., Nicolson A., Gleeson F.V. (2022). Developing an understanding of artificial intelligence lung nodule risk prediction using insights from the Brock model. Eur. Radiol..

[29] Venkadesh K.V., Setio A.A.A., Schreuder A., Scholten E.T., Chung K., W Wille M.M., Saghir Z., van Ginneken B., Prokop M., Jacobs C. (2021). Deep Learning for Malignancy Risk Estimation of Pulmonary Nodules Detected at Low-Dose Screening CT. Radiology.

[30] Godfrey C.M., Shipe M.E., Welty V.F., Maiga A.W., Aldrich M.C., Montgomery C., Crockett J., Vaszar L.T., Regis S., Isbell J.M. (2023). The Thoracic Research Evaluation and Treatment 2.0 Model: A Lung Cancer Prediction Model for Indeterminate Nodules Referred for Specialist Evaluation. Chest.

[31] Wu Z., Huang T., Zhang S., Cheng D., Li W., Chen B. (2021). A prediction model to evaluate the pretest risk of malignancy in solitary pulmonary nodules: Evidence from a large Chinese southwestern population. J. Cancer Res. Clin. Oncol..

[32] Chen K., Nie Y., Park S., Zhang K., Zhang Y., Liu Y., Hui B., Zhou L., Wang X., Qi Q. (2021). Development and Validation of Machine Learning-based Model for the Prediction of Malignancy in Multiple Pulmonary Nodules: Analysis from Multicentric Cohorts. Clin. Cancer Res..

[33] Gao R., Li T., Tang Y., Xu K., Khan M., Kammer M., Antic S.L., Deppen S., Huo Y., Lasko T.A. (2022). Reducing uncertainty in cancer risk estimation for patients with indeterminate pulmonary nodules using an integrated deep learning model. Comput. Biol. Med..

[34] Baldwin D.R., Gustafson J., Pickup L., Arteta C., Novotny P., Declerck J., Kadir T., Figueiras C., Sterba A., Exell A. (2020). External validation of a convolutional neural network artificial intelligence tool to predict malignancy in pulmonary nodules. Thorax.

