# Supplementary material for: Lung Cancer Risk Prediction in Patients with Persistent Pulmonary Nodules Using the Brock Model and Sybil Model
Source: Cancers (Basel). 2025 Apr 29;17(9):1499. doi: 10.3390/cancers17091499 (PMC12070823; doi:10.3390/cancers17091499)
Supplement: Supplementary file 1 [file cancers-17-01499-s001.zip › cancers-3562947-supplementary/Supplementary Table S1.pdf]

Table S1. Comparison of sensitivity, specificity, positive predictive value (PPV), negative predictive value (NPV) across the Brock, Sybil and Logistic Regression (LR) models.

Model	AUC (95% CI)	Threshold	Sensitivity (95% CI)	Specificity (95% CI)	PPV (95% CI)	NPV (95% CI)
**Brock model**	0.679 (0.595-0.763)	0.05	0.968 (0.890–0.991)	0.000 (0.000–0.016)	0.201 (0.159–0.250)	0.000 (0.000–0.658)
		0.10	0.968 (0.890–0.991)	0.004 (0.001–0.023)	0.201 (0.160–0.251)	0.333 (0.061–0.792)
		0.15	0.968 (0.890–0.991)	0.008 (0.002–0.030)	0.202 (0.160–0.251)	0.500 (0.150–0.850)
		0.20	0.935 (0.846–0.975)	0.008 (0.002–0.030)	0.197 (0.155–0.246)	0.333 (0.097–0.700)
**Sybil model**	0.666 (0.597-0.740)	0.05	0.468 (0.349–0.590)	0.787 (0.730–0.834)	0.363 (0.266–0.472)	0.851 (0.798–0.892)
		0.10	0.339 (0.233–0.463)	0.803 (0.748–0.849)	0.309 (0.212–0.426)	0.824 (0.770–0.868)
		0.15	0.194 (0.114–0.309)	0.900 (0.855–0.932)	0.333 (0.202–0.497)	0.811 (0.760–0.854)
		0.20	0.194 (0.114–0.309)	0.912 (0.869–0.942)	0.364 (0.222–0.534)	0.813 (0.762–0.856)
**LR model***	0.729 (0.597-0.861)	0.05	0.913 (0.732–0.976)	0.221 (0.138–0.333)	0.284 (0.194–0.395)	0.882 (0.657–0.967)
		0.10	0.826 (0.629–0.930)	0.426 (0.316–0.545)	0.328 (0.221–0.456)	0.879 (0.727–0.952)
		0.15	0.783 (0.581–0.903)	0.662 (0.543–0.763)	0.439 (0.299–0.590)	0.900 (0.786–0.957)
		0.20	0.739 (0.535–0.875)	0.721 (0.604–0.813)	0.472 (0.320–0.630)	0.891 (0.782–0.949)

Sensitivity is the proportion of malignant nodules correctly identified as malignant. Specificity is the proportion of benign nodules correctly identified as benign. PPV is the proportion of positive predictions that are malignant nodules. NPV is the proportion of negative predictions that are benign nodules. *The LR model was chosen based on 5-fold cross-validations using the training data (70%), the model was evaluated in the 30% hold-out testing data not used for model development.
