# Supplementary figures and images for: Lung Cancer Risk Prediction in Patients with Persistent Pulmonary Nodules Using the Brock Model and Sybil Model
Source: Cancers (Basel). 2025 Apr 29;17(9):1499. doi: 10.3390/cancers17091499 (PMC12070823; doi:10.3390/cancers17091499)

**A**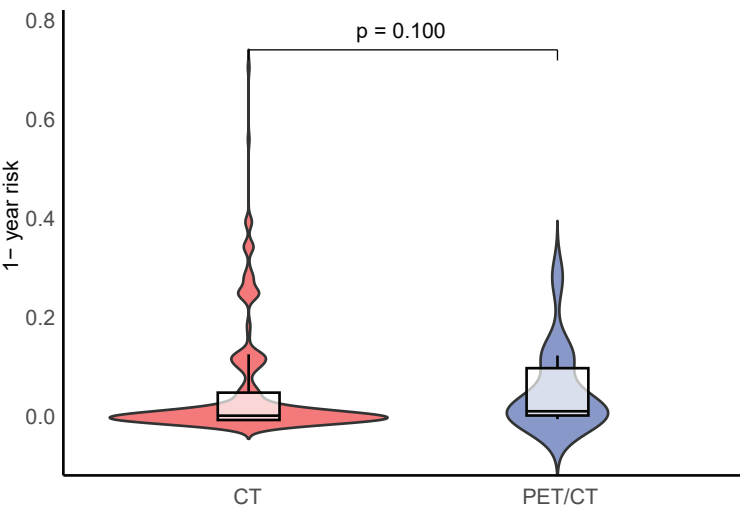**B**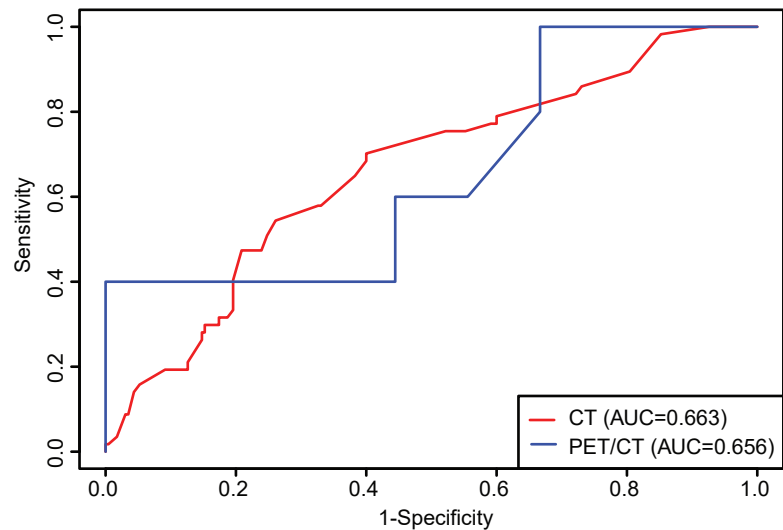**C**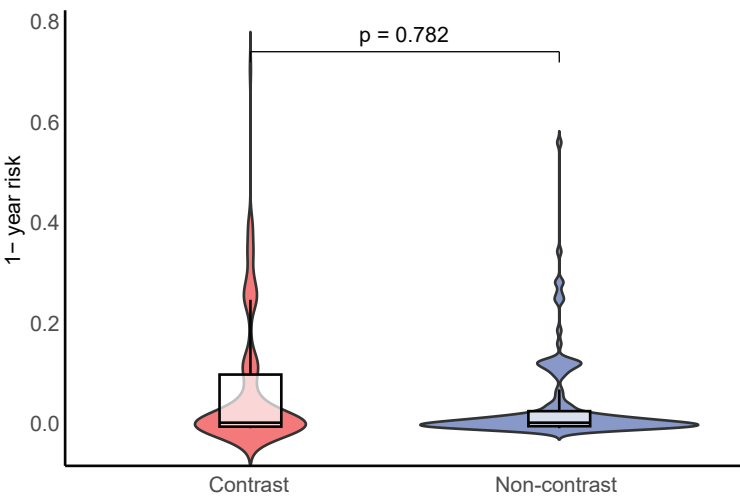**D**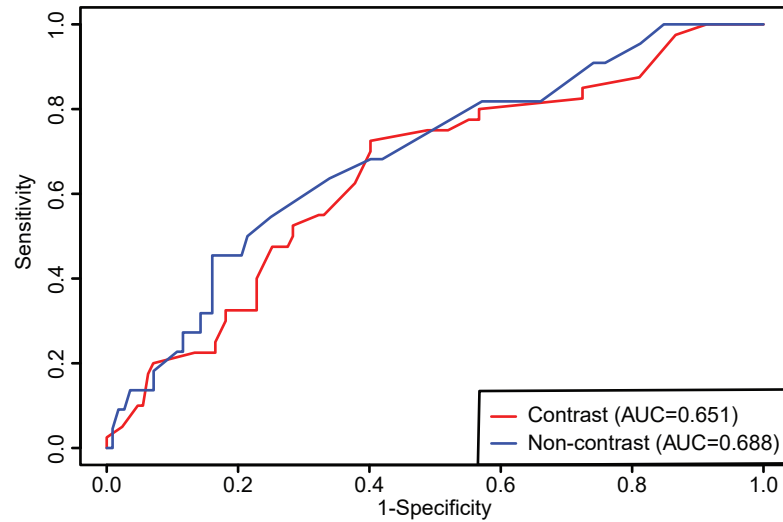

Supplement: Supplementary file 1 [file cancers-17-01499-s001.zip › cancers-3562947-supplementary/Supplementary Figure S1.pdf]
